# Spinocerebellar ataxia: an update

**DOI:** 10.1007/s00415-018-9076-4

**Published:** 2018-10-03

**Authors:** Roisin Sullivan, Wai Yan Yau, Emer O’Connor, Henry Houlden

**Affiliations:** Department of Neuromuscular Diseases, UCL Queen’s Square Institute of Neurology, Queen’s Square House, Queen’s Square, London, WC1N 3BG UK

**Keywords:** Spinocerebellar ataxia, Molecular diagnosis, Next-generation sequencing

## Abstract

Spinocerebellar ataxia (SCA) is a heterogeneous group of neurodegenerative ataxic disorders with autosomal dominant inheritance. We aim to provide an update on the recent clinical and scientific progresses in SCA where numerous novel genes have been identified with next-generation sequencing techniques. The main disease mechanisms of these SCAs include toxic RNA gain-of-function, mitochondrial dysfunction, channelopathies, autophagy and transcription dysregulation. Recent studies have also demonstrated the importance of DNA repair pathways in modifying SCA with CAG expansions. In addition, we summarise the latest technological advances in detecting known and novel repeat expansion in SCA. Finally, we discuss the roles of antisense oligonucleotides and RNA-based therapy as potential treatments.

**Electronic supplementary material:**

The online version of this article (10.1007/s00415-018-9076-4) contains supplementary material, which is available to authorized users.

## Introduction

**Fig. 1:**
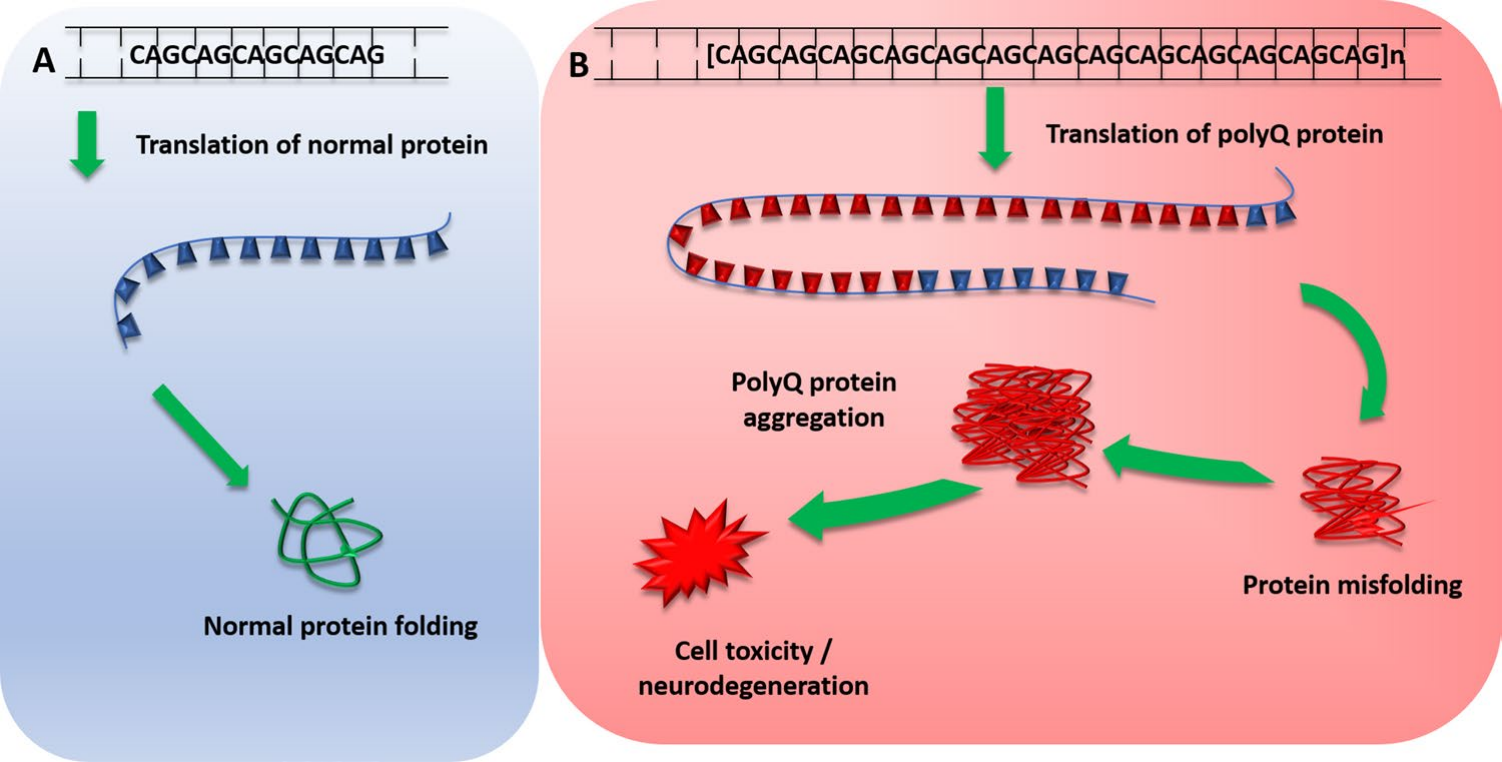
Mechanism of polyglutamine protein expansion repeats. **a** Normal translation of polyglutamine repeat within normal repeat range, producing normal protein transcript and protein folding. **b** Pathogenic polyglutamine expansion repeat length leads to translation of expanded abnormal PolyQ repeat, which leads to protein misfolding. Misfolded polyQ proteins form aggregates which lead to various cellular process dysfunctions, leading to cell toxicity and degeneration. *PolyQ* polyglutamine proteins

The Spinocerebellar ataxias (SCA) are a subset of hereditary cerebellar ataxias that are autosomal dominantly transmitted. They are progressive neurodegenerative diseases that share the clinical features of ataxia, which arise from the progressive degeneration of the cerebellum but can also affect other connected regions, including the brain stem. They are a highly heterogenous group of disorders with a complex genotype–phenotype spectrum; many SCAs are caused by CAG nucleotide repeat expansions that encode polyglutamine, and therefore, involve the toxic polyglutamine protein (polyQ) (Fig. 1) [1]. Recent advances in next-generation sequencing have identified new genes implicated in SCAs providing insights into disease transmission and pathogenesis. Here, we discuss updates in epidemiology, clinical features, molecular mechanisms and their potential implications in the future.

## What is new in the epidemiology of SCA and its subtypes?

A recent systemic review shows that the global prevalence of SCA is 3 in 100,000 [2], however, a wide regional variation exists. SCA3 is commonest subtype around the globe [3–5], SCA2 is more prevalent in Cuba than SCA3 whilst SCA7 is the most frequent subtype in Venezuela due to strong founder’s effect [6, 7]. SCA6 is one of the most common ADCA in the North of England, with a global prevalence of 5.2/100,000 [8]. There are various mutations described in SCA, although repeat expansions still account for almost half of SCA diagnosis in European cohort. In 412 undiagnosed autosomal dominant cerebellar ataxia (ADCA) without known repeat expansion, 59 individuals (14.3%) were found to harbor pathogenic variants [9]. Thirty five of these variants (8.5%) belong to channel genes. In contrast, conventional mutations in channel genes are rare in Han Chinese cohort [10]. In another cohort of 194 individuals with undiagnosed ADCA, SCA14 accounts for 6.7% of the studied population [11]. Other similar studies in Germany, United Kingdom, France, United States, Japan and Taiwan confirm the relative rarity of SCA 8, 23, 35, 36 and 42. They are each responsible for less than 1% of undiagnosed ADCA [12–18] although the advance in diversity genetics will further reveal the frequency of these genes in other populations.

## Make sense of SCA clinical features

**Fig. 2:**
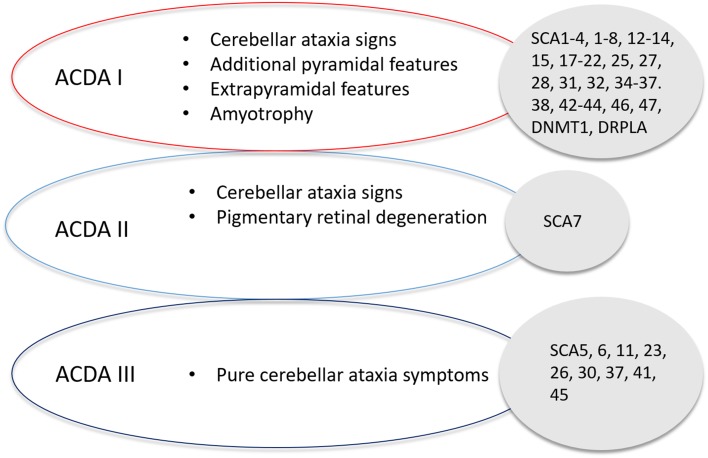
Harding’s classification of Spinocerebellar Ataxia, detailing the classification of SCA based on symptom presentation and the associated SCAs with that classification

**Fig. 3:**
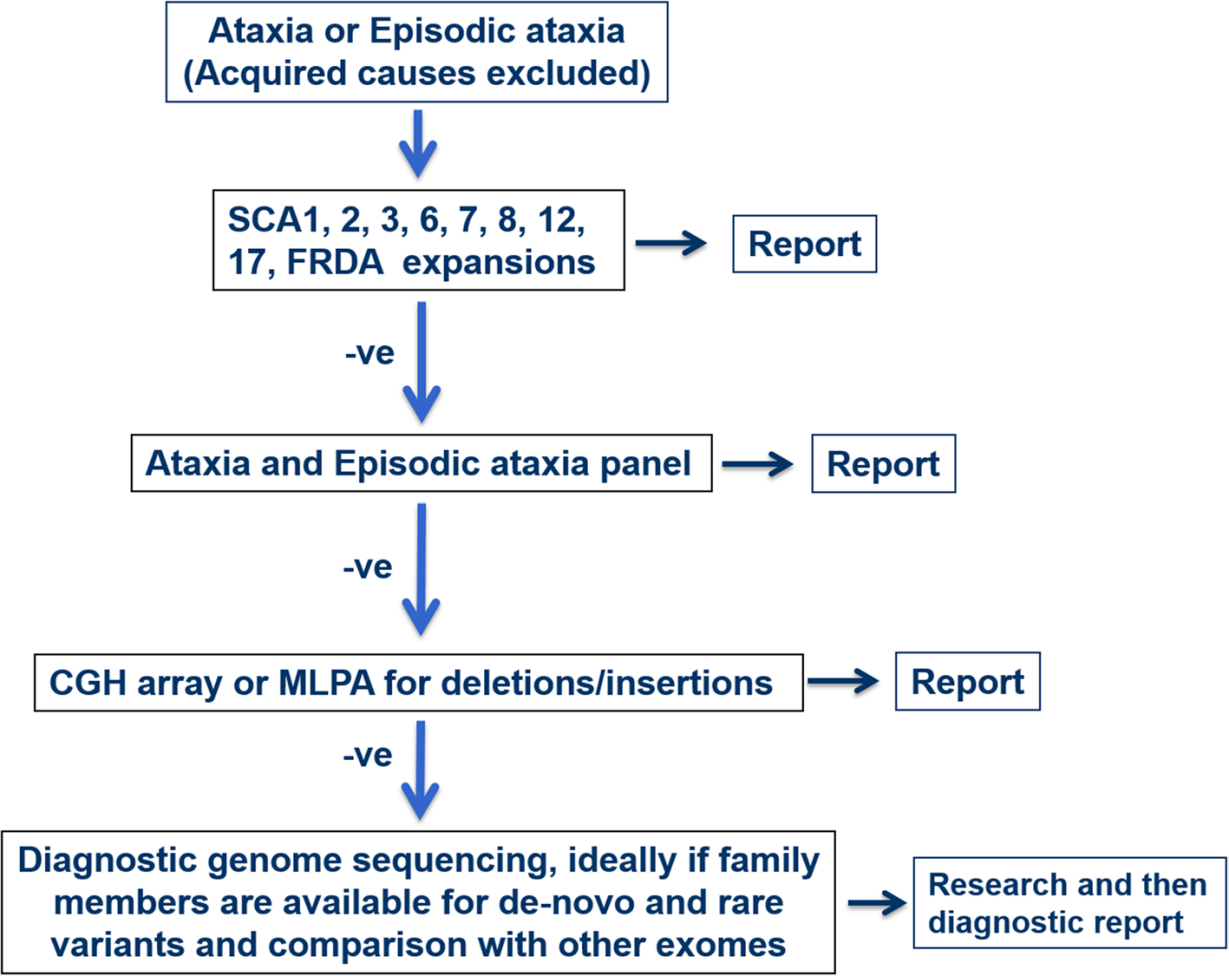
Flowchart of diagnosis pathway based on either positive or negative result of each diagnostic test. −*ve—negative*

The core triad of symptoms of SCAs include gait ataxia and incoordination, nystagmus/visual problems and dysarthria. Patients can present with additional features such as pyramidal, extrapyramidal signs, ophthalmoplegia and cognitive impairment in specific SCAs. Harding’s classification of ADCA in 1982 is still useful in the clinical setting (Fig. 2) [19]. ADCA type 1 describes cerebellar ataxia with variable additional signs. This list is ever expanding and includes SCA1–4, 8, 10, 12–14, 15, 17–22, 25, 27, 28, 31, 32, 34–37, 38, 42–44, 46, 47, ataxia with DNMT1 and DRPLA [20–23]. ADCA type 2 describes cerebellar ataxia with pigmentary macular degeneration and consists of only SCA 7 [20]. ADCA type 3 refers to ‘pure’ cerebellar ataxia, which includes SCA 5, 6, 11, 23, 26, 30, 37, 41 and 45 [20, 24]. Several SCAs have characteristic clinical features in addition to cerebellar ataxia and helps distinguish them from other subtypes. For instance, SCA 12, 15 and 27 have upper limb postural tremor [25–27]; SCA 14 may have myoclonus and task-specific dystonia [28]; and a subset of SCA 36 have facio-lingual fasciculation with sensorineural hearing loss [29]. Table 1 shows a non-exhaustive list of distinctive clinical signs that feature prominently with cerebellar ataxia, adapted from a recent systematic review [30]. International Parkinson and Movement disorders Task Force recently proposes a new classification of SCA, dividing them into pure or relatively pure ataxia and complex ataxia, which overlaps with above mentioned ADCA classification [31]. A phenotype-first approach remains pertinent in molecular diagnosis of rare genetic disorders (Fig. 3) [32]. Clinicians should also consider genetic testing for primary episodic ataxias (EA), especially with history of episodic attacks of imbalance, dysarthria, vertigo and/or diplopia lasting hours–days. EAs are autosomal dominant channelopathies and they mostly manifest before age 20 years [33]. They can be associated with other paroxysmal neurological disorders such as migraines, epilepsy and dystonia. Patients with EA type 1 also have interictal myokymia. However, progressive cerebellar ataxia may also occur in a proportion of patients with non-expansion mutations in *KCNA1* and *CACNA1A*, especially later in the disease course [34, 35]. Unfortunately, the utility of clinical–genetic classification in SCA is limited by high level of phenotype–genotype overlap.

**Table 1:** SCA subtypes with associated clinical signs that feature prominently with cerebellar ataxia

Associated clinical features	Genetic subtypes
Peripheral neuropathy	1, 2, 3, 4, 18, 25, 38, 43, 46
Pyramidal signs	1, 3, 7, 8, 10, 14, 15, 17, 35, 40, 43
Dystonia	3, 14, 17, 20, 35
Myoclonus	14
Parkinsonism	2, 3, 10, 14, 17, 19/22, 21
Tremor	12, 15, 27
Chorea	17, 27, DRPLA
Cognitive impairment	2, 8, 13, 17, 19/22, 21, 36, 44, DRPLA
Psychiatric symptoms	2, 17
Ophthalmoplegia	2, 3, 28, 40
Visual impairment	7
Face/tongue fasciculation	36
Ichthyosiform plaques	34
Seizures	10, 19/22, ATN1
Narcolepsy	DNMT1
Hearing loss	31, 36, DNMT1

*ATN1* atrophin 1, mutation responsible for dentatorubral–pallidoluysian atrophy, *DNA methyltransferase 1*, mutation responsible for ADCA-deafness and narcolepsy

## Advances in molecular diagnoses and disease mechanisms

Advances in next-generation sequencing (NGS) have facilitated further insights into the molecular causes of SCA. When NGS becomes translated to clinical practise, it has the potential to increase the success of molecular diagnosis for patients currently undiagnosed by standard genetic testing. Recent successes include conventional mutations in CCDC88C, TRPC3, CACNA1G, MME, GRM1, FAT2, PLD3 and PUM1 [23, 24, 36–40] since the last update in this journal in 2015 (see Table 2) [41]. Exome sequencing has an average diagnostic rate of 36% compared to target resequencing panel of 17% [42]. Exome sequencing identifies less classical phenotype–genotype correlations and detects new mutations in known cerebellar genes [43]. We outline the roles of toxic RNA gain-of-function, mitochondrial defects, channelopathy, autophagy and transcriptional dysregulation in pathogenesis of SCA.

**Table 2:** Summary of major clinical characteristics of novel SCA genes described since 2015

Gene/locus	Mutation	Number of pedigrees	Clinical features	Pathogenic mechanisms
CCDC88C (SCA 40) [36]	Missense c.1391G>A (pR464H)	4 Probands from 1 family	Cerebellar ataxia, hyperreflexia	JNK pathway hyperphosphorylation induced cellular apoptosis
TRPC3 (SCA 41) [37]	Missense c.2285G>A (pR762H)	1 Proband from 1 family	Cerebellar ataxia	Toxic gain-of-function, channelopathy
CACNA1G (SCA 42) [38, 57]	Missense c.5144G>A (pR1715H)	30 Probands from 5 families	Cerebellar ataxia	Haplo-insufficiency of T-type calcium channel
MME (SCA 43) [39]	Missense c.428G>A (p.C143Y)	7 Probands from 1 family	Cerebellar ataxia with peripheral neuropathy	Haplo-insufficiency of neprilysin, a zinc-dependent metalloproteinase
GRM1 (SCA 44) [40]	Missense c.2375A>G (p.Y792C) c.785A>G (p.Y262C)	7 Probands from 2 families	Cerebellar ataxia with pyramidal sign	Toxic gain-of-function metabotropic glutamate receptor 1
FAT2 (SCA 45) [24]	Missense c.10946G>A (p.R3649Q) c.10758G>C (p.K3586N)	6 Probands from 1 family	Cerebellar ataxia	?affect cell adhesion
PLD3 (SCA 46) [24]	Missense c.923T>C (L308P)	11 Probands from 1 family	Cerebellar ataxia with peripheral neuropathy	Haplo-insufficiency of phospholipase D activity
PUM1 (SCA 47) [23]	Missense g.31414862 T>A (p.T1035S)	9 Probands from 1 family	Cerebellar ataxia	Haplo-insufficiency of PUM1

*CCDC88C* coiled-coil domain containing 88C, *JNK* c-Jun N-terminal kinase, *TRPC3* transient receptor potential cation channel subfamily C member 3, *CACNA1G* voltage sensor S4 segment of domain IV in Cav3.1T-type calcium channel protein MME neprilysin, *GRM1* glutamate metabotropic receptor 1, *FAT2* FAT atypical cadherin 2, *PLD3* phospholipase D3, *PUM1* RNA-binding protein Pumilio1

### Toxic RNA gain-of-function

**Fig. 4:**
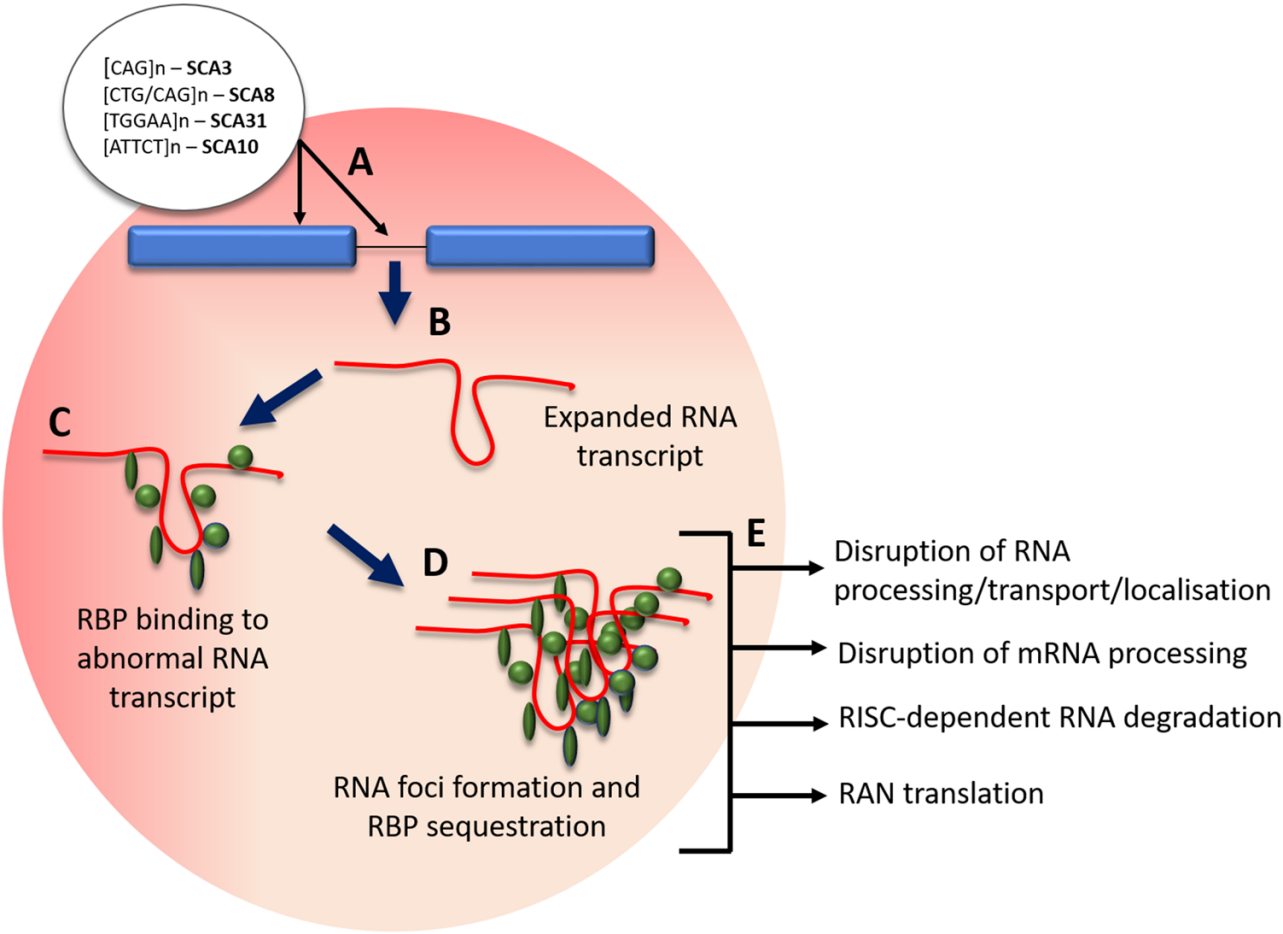
Mechanism of RNA foci formation and effects. **a** Pathogenic SCA intronic and exonic expansion repeats; **b** transcription of expanded repeat into expanded mRNA/pre-RNA; **c** binding of regulatory binding proteins (RBP) to abnormal mRNA transcript; **d** RBP protein sequestration and abnormal transcript aggregation; **e** effects of RBP sequestration on cellular processes. *RBP* regulatory binding proteins, *RAN* repeat-associated non-ATG

Non-coding repeat expansions are implicated in SCA subtypes that include SCA 8, 10, 12, 31, 36 and 37. The hallmarks are transcribed nuclear accumulations of repeat RNA-binding proteins that can cause RNA toxicity and lead to disease pathogenesis (see Fig. 4) [44]. An intronic ATTCT pentanucleotide repeat expansion in *ATXN10* has been found to cause SCA10, with pathogenicity in the range of 800–4500 repeats [45]. Cytoplasmic and nuclear foci form in SCA10 cells and SCA10 transgenic mice brain from degradation-resistant, aggregated AUUCU RNA, which is formed from the splicing out of intron 9 from *ATXN10* pre-mRNA. The expanded AUUCU RNA binds to heterogeneous nuclear ribonucleoprotein K (hnRNPK), a spicing factor, causing its sequestration and loss of function. Ultimately this leads to the mitochondrial accumulation of protein kinase C δ (PKCδ) and apoptosis of SCA10 cells [46]. An intronic GGCCTG hexanucleotide repeat expansion was found in the gene NOP56 using genome-wide linkage, responsible for SCA 36. Patients’ lymphoblastoid cells contain RNA foci, and transcription of MIR1292, a neighbouring miMRA is reduced. These implicate a toxic RNA gain-of-function pathological mechanism in SCA 36 [29]. A toxic gain of function effect was found to be implicated in SCA8 pathogenesis, with the CTG CAG repeat expansion, which is bidirectionally expressed, causing [CUG]n transcript accumulation of ribonuclear inclusions that localise with the RNA binding protein Mbnl1. The downstream effects and alternative splicing contribute to the movement disorder phenotype in a SCA8 mice model [47]. Bidirectional expression of sense [CUG]n and antisense [CAG]n has been found in other disorders such as at the DM1 locus [48] and in HDL2, which also forms ribonuclease inclusions [49]. Most recently an unstable intronic ATTTC repeat has been identified as the pathogenic cause of SCA37 in two Spanish cohorts, dysregulating reelin adaptor protein disabled-1 coding *DAB1* expression, leading to alternative splicing, an RNA switch and an upregulation of reelin-DAB1 signalling in the SCA37 cerebellum [50].

### Mitochondrial dysfunction

Recently advances have been made in the understanding of mitochondrial dysfunction and ataxia, with several genes related to mitochondrial function identified. These include mutations in OPA1, a mitochondrial dynamin-like GTPase and POLG [9], coding for the catalytic subunit of mitochondrial DNA polymerase gamma, as well as MTATP6 [22]; both of which involve mitochondrial dysfunction. Functional mitochondria are required for all cell processes including cell proliferation, differentiation, apoptotic cell death and are a crucial part of signalling cascades [51]. Findings have suggested that mitochondrial dysfunction and reactive oxygen species (ROS) may be implicated in SCA2 pathogenesis, with an increase in mitochondrial oxidative stress in SCA2 patient fibroblasts, as well as changes in mitochondrial respiratory chain (MRC) enzymes and in mitochondrial morphology. Importantly these effects were increased in a SCA2 fibroblast line with an expanded CAG repeat from a patient yet to exhibit clinical symptoms, suggesting that mitochondrial dysfunction may precede disease onset. Furthermore, the antioxidant coenzyme Q10 (CoQ10) was found to improve oxidative stress in fibroblasts [52]. Oxidative damage is a frequent feature of neurodegenerative conditions, including Alzheimer’s disease and Parkinson’s disease, due to the brains high oxygen utilisation and high content of oxidisable polyunsaturated fatty acids [53]. The polymorphic locus A100398G has been implicated in early age of onset in Cuban SCA2 patients [54] and has recently been described in a more severe cognitive phenotype in a 42 SCA2 cohort, corroborating with prior findings in the Cuban cohort [55].

### Channelopathies

Mutations coding for ion channel subunits or regulatory proteins, channelopathies, are frequently involved in the pathology of SCAs. Inherited channelopathies can alter ion channel function by mechanisms including inhibiting the ion movement through an open channel pore and altering ion channel gating through changes to channel opening processes or inactivation processes [56]. A recent study using amplicon-based panel sequencing for 65 genes on 412 index patients found that channelopathies (with mutations in CACNA1A, CACNA1G, KCND3 and KCNC3 in particular) had the earliest age of onset, longest disease duration and slowest disease progression, as well as purest cerebellar presentation. Interestingly they found a prominent implication of SPG7 and CACNA1A in ataxic point mutations [9].

In fact, several newly described SCA gene mutations (GRM1, CACNA1G, TRPC3) are implicated on physiological functions of the cellular channels [37, 38, 40, 57]. Transient receptor potential C3 (TRPC3) mutation occurs in a highly conserved region of the nonselective cation channel and is proposed to affect regulation of channel gating [37]. It has only been described in a sporadic case of adult-onset cerebellar ataxia. Calcium voltage-gated channel subunit alpha 1 G (CACNA1G) mutation leads to altered physiological property in the low voltage-gated calcium channel and causes a relatively pure cerebellar ataxia [38, 57]. CACNA1G channels are most prominently expressed in Purkinje cells and deep cerebellar nuclei. Electrophysiological study demonstrates that the mutation shifts the current–voltage and the steady-state activation curves of the mutant transfected cells positively [57]. In silico this causes decreased neuronal excitability. Glutamate metabotropic receptor 1 (GRM1) gain-of-function mutations result in excessive glutamate receptor signalling and intracellular calcium level [40]. The authors hypothesised excitotoxicity in cerebellar Purkinje cells as the cause of ADCA in two families.

### Autophagy

Autophagy is one of the main pathways for degradation of misfolded proteins; the other is the ubiquitin–proteasome system. Autophagy has been linked to neurodegeneration [58]. Both of SCA 3 fibroblast and SCA 7 mouth model demonstrate impaired autophagy [59, 60]. A mammalian target of rapamycin (mTOR) inhibitor that upregulates autophagy clears ataxin-3 and aggregates in brain in a SCA 3 mouse model. This appears to slow motor deterioration [61]. Until recently, it remains unclear whether the misfolded protein accumulation causes the dysfunction of autophagy or whether impaired autophagy leads to accumulation of misfolded protein. Ashkenazi et al. demonstrate that wide-type ataxin-3 polyglutamine repeat interacts with beclin 1, a key initiator of autophagy [62]. This interaction permits the deubiquitinase activity of ataxin-3 to protect beclin 1 from proteasome-mediated degradation. Thus, it allows autophagy to occur. Long polyglutamine expansion in mutant ataxin-3 competes with wide type ataxin-3 interaction with beclin 1 and leads to impaired starvation-induced autophagy. This highlights the direct role abnormally repeat expansion plays in neurodegeneration of SCA other than protein aggregation.

### Transcriptional dysregulation

The molecular mechanisms of number SCAs (SCA 1, 2, 3, 7, 17) involve interference with transcription through different mechanisms [63]. These include protein–DNA interactions, acetylation, phosphorylation and RNA interference. The mutant protein Ataxin-1, involved in SCA1 pathogenesis, is a transcription activator, whilst the polyglutamine expansion of SCA 17 occurs within the TATA box-binding protein (TBP), an essential transcription factor [64, 65]. A recently described mutation in RNA-binding protein Pumilio (PUM1) results in ADCA, coined SCA 47 [23]. Experiments in patients’ fibroblasts demonstrated that PUM1 protein acts as atranscription repressor. Reduced expression of PUM1 suppresses dendritic arborization. Interestingly, the phenotypic severity of PUM1 mutation varies with the degree which the missense mutations/deletion reduce PUM1 protein levels. Around 50% reduction of PUM1 protein leads to a severe syndromic development delay whilst 25% reduction produces adult-onset cerebellar ataxia.

## Genetic modifiers

The clinical diversity of the hereditary cerebellar ataxias in terms of age of onset, progression and severity of disease, strongly suggests the presence of modifying factors. We are beginning to dissect the complex mechanisms of genetic modifiers through the delineation of molecular pathways in the era of high-throughput sequencing. Common genetic variants, with a significant effect size, may act as genetic modifiers in rare mendelian conditions such as SCAs. A Genome-Wide Association Studies (GWAS) on Huntington Disease (HD), a (CAG)n expansion disorder, identified three significant loci with enrichment in DNA repair networks [66]. Subsequently, genotyping of these single nucleotide polymorphisms (SNPs) in 1462 subjects with CAG repeat SCAs and HD showed a significant association between DNA repair genes and the age at onset of SCAs and HD, with SNPs in FAN1 and PMS2 reaching the lowest *p* values [67]. FAN1 is a repair nuclease that is recruited to sites of crosslink damage and PMS2 endonuclease is a mismatch repair (MMR) protein [68, 69]. In another association study of 137 parent–child transmissions in SCA 3, a variant ERCC6 (Cockayne syndrome protein CSB) is associated with an expansion bias of (CAG)n [70]. Tight DNA repair regulation is an integral process to maintain integrity of expansions in replication and translation [71]. Dysregulation of DNA repair genes is postulated to result in somatic expansions in non-expanding cells of SCAs with trinucleotide repeat expansions and subsequent disease progression. An International GWAS of repeat expansion ataxia will be extremely valuable to provide further insights into the genetic factors influencing the clinical characteristics of these disorders.

## Advances in diagnosis of repeat expansion disorders

The vast heterogeneity of SCA highlights the effectiveness of whole-exome and -genome sequencing (WES/WGS) as a diagnostic tool. Multiple reads are required for SCAs with conventional disease-causing mutations including single-nucleotide polymorphisms (SNPs), deletions and insertions, to span the full length of the nonreference allele [72]. Currently, high-throughput sequencing technologies are limited to read lengths of approximately 150 base pairs (bp), however, pathogenic expansion repeats can span to thousands of bp in size, therefore, being unidentifiable by short-read sequencing technologies.

Repeat primed-PCR and subsequent fragment analysis is widely used to detect repeat expansions, such as the repeat expansion in the *C9orf72* locus implicated in both Amyotrophic Lateral Sclerosis (ALS) and Frontotemporal Dementia (FTD). However, these approaches have a frequent risk of misinterpretation due to false positives and negatives because of indels in the repeat flanking regions and variability in diagnostic laboratory protocols [73]. In addition, repeat length size cannot be estimated by these techniques. The current gold standard for estimating repeat length is Southern blotting which requires substantial amounts of DNA (approximately 10 µg) and is vulnerable to somatic heterogeneity, reducing the precision of size estimation [74]. A novel method named tethering PCR, has recently been proposed by Cagnoli et al. to identify pathogenic expansions and estimate repeat size in SCA1, 2, 3, 6 and 7, as well as recognise large alleles and repeat interruptions, negating the need to perform a secondary test (i.e. Southern blot) [75].

Long-read sequencing technologies such as the Oxford Nanopore sequencing and PacBio single-molecule real-time (SMRT) facilitate the sequencing of more than 10,000 bp DNA sequence lengths. Nanopore sequencing uses a protein nanopore covalently attached to an adaptor molecule to identify unlabeled nucleoside 5′ monophosphate molecules. It has an average accuracy of 99.8%. It can function without exonuclease and has a high accuracy of not registering the same nucleotide twice due to translocation through the nanopore [76]. By contrast, SMRT sequencing uses real-time imaging of fluorescently tagged nucleotides during DNA synthesis along template models, using DNA polymerase as a reaction driver [77]. PacBio reads have around a 15% average higher error rate [78], however, Oxford Nanopore platforms also have limitations with their MiniION sequencer having a reported estimated base-calling error rate of 38.2% [79].

Due to the popularity of WGS, developing a method for pathogenic repeat expansions has been of interest. Recently a software package called ExpansionHunter has been developed by Illumina that can determine the size of repeats of varying size, including very large pathogenic expansions much longer than the read length, using PCR-free WGS data [80]. They developed an algorithm able to identify reads in several different conditions; reads that span the full length, reads that fully contain the repeat [‘in-repeat’ reads (IRR)] and repeats that include the repeat and flanking sequence on one side of the repeat. The software correctly classified all *C9orf72* expanded samples in an ALS cohort as either expanded, possibly expanded or wild-type as well as 8 other pathogenic repeat expansions, including samples from SCA1 and SCA3 cohorts. The tool facilitates the screening of repeat expansions using a single run of WGS, with PCR-free WGS data. Development is still in progress as currently the software requires an STR to be specified by reference coordinate and repeat motif. However, future association studies and available WGS-data will provide a genome-wide STR database, which will greatly enhance the utility of ExpansionHunter.

## Future direction and potential treatments

Advancements in the understanding of pathophysiologic mechanisms facilitate the potential to find new therapeutic targets. Current treatment pipelines involve the use of pharmacological molecules to target affected downstream pathways, as well as genetic therapies to decrease toxic polyQ gene products. The former could benefit a larger proportion of patients if the targeted pathway is implicated in several neurodegenerative diseases, however, if a mutant protein affects multiple cellular processes it could prove difficult to target a vital pathway to provide effective treatment [81].

### Antisense oligonucleotides

Antisense oligonucleotides (ASOs) are small single-stranded sequences of DNA that have the gene-targeting effect of reducing levels of toxic protein or making non-toxic modifications which are valuable tools for neurodegenerative diseases [82]. Successful uptake of ASOs facilitates their gene-modulating effects and relies on effective delivery. Mechanisms include lysosomal or endosomal compartmental internalisation, association with high- and low-binding plasma proteins and cellular trafficking.

ASOs decrease the expression of the target protein using Watson–Crick hybridization to bind to complementary mRNA transcripts and recruit RNase H enzymes [83]. ASO-mediated exon skipping has been successfully applied to SCA3 fibroblasts, removing the central 88 amino acid region of the ataxin-3 protein in one study to halt production of potentially toxic cleavage fragments; however, protein-modification effect was at a low-level [84, 85]. ASO therapy targeting SCA3 mice models has also been shown to reduce disease protein levels by greater than 50% in the cerebellum, diencephalon, cervical spinal cord and forebrain, presenting with no signs of microgliosis and astrogliosis, indicating its potential for a well-tolerated preventative therapy despite no observable reduction in motor phenotype [86, 87]. ASO therapy targeting ATXN2 has shown slowed progression of the motor phenotype and improved survival in both SCA2 [81] and ALS transgenic mouse model TDP-43 [88].

### RNA based therapy

Therapeutics based on RNA interface (RNAi) harness the cellular mechanism of gene expression silencing to reduce the expression of pathological proteins. Synthetic small interfering RNA (siRNAs) and short hairpin RNA (shRNAs) control the RNAi process of target mRNA enzymatic cleavage in a predictable and consistent action [89]. RNAi has been successfully used to reduce mutant ataxin-7 in SCA7 mouse models in a nonallele- [90] and allele-specific manner [91]. ATXN3 has also been suppressed in Machado–Joseph disease (MJD) mouse models, with reports of improved motor symptoms and neuropathology [92, 93].

### Stem cell based therapy

Some stem cell-based therapies have been performed on several cerebellar mutant mice, such as SCA1 mouse models [94–98], with reports of normalized motor deficits and reduced Purkinje cell loss and SCA2 models which showed delayed onset of motor function deterioration [99]. Phase I and II clinical trials, one of which involved intravenously infused human umbilical cord mesenchymal stem cell (UC-MSC) into SCA1, 2 or 3 cohorts (*n* = 16) reported no transplantation side effects and an improved International Cooperative Ataxia Rating Scale (ICARS) and Berg Balance Scale (BBS) score after 6 months post-transplantation [100]. Another trial on 14 SCA patients using intrathecal injection of UC-MSC reported significant ICARS and Activity of Daily Living Scale (ADL) scores, which decreased after 1 month of treatment, although 8 patients remained stable for 6–9 months post-transplantation [101]. These suggest a potential proof of principle for stem cell therapy as a therapeutic intervention, however, assessment of efficacy and safety requires further clinical trials.

## Conclusions

Tremendous scientific progress has occurred in the understanding of spinocerebellar ataxia. Next-generation sequencing has helped improve the diagnostic accuracy of SCAs and discover new disease mechanisms. New technologies such as nanopore and ExpansionHunter may help improve diagnosis of known and new SCAs with repeat expansions in future. As outlined, evidence suggests that genes in DNA repair pathways appear to play a modifying role. An international GWAS of repeat expansion ataxia would be worthwhile to pursue these potential therapeutic targets. Meanwhile, emerging therapies for neurogenetic diseases such as ASOs also provide physicians and patients of SCA hopes of effective treatments in the near future.

## Electronic supplementary material

Below is the link to the electronic supplementary material.

Supplementary material 1 (DOCX 56 KB)
