# Supplementary material for: Spinocerebellar ataxia: an update
Source: J Neurol. 2018 Oct 3;266(2):533–44. doi: 10.1007/s00415-018-9076-4 (PMC6373366; doi:10.1007/s00415-018-9076-4)
Supplement: Supplementary file 1 — Supplementary material 1 (DOCX 56 KB) [file 415_2018_9076_MOESM1_ESM.docx]

| *Disease Subtype* | *Gene/Locus* | *Reference* |
| --- | --- | --- |
| SCA1 | ATXN1 | (102) |
| SCA2 | ATXN2 | (103) |
| SCA3 | ATXN3 | (104) |
| SCA4 | 16q22.1 | (105) |
| SCA5 | SPTBN | (106) |
| SCA6 | CACNA1A | (107) |
| SCA7 | ATXN7 | (108) |
| SCA8 | ATXN8 | (109) |
| SCA9 | Not assigned |  |
| SCA10 | ATXN10 | (110) |
| SCA11 | TTBK2 | (111) |
| SCA12 | PPP2R2B | (25) |
| SCA13 | KCNC3 | (112) |
| SCA14 | PRKCG | (113) |
| SCA15 | ITPR1 | (114) |
| SCA16 | ITPR1 | (115) |
| SCA17 | TBP | (116) |
| SCA18 | 7q22-q32 | (117) |
| SCA19/22 | KCND3 | (118) |
| SCA20 | 11q12 | (119) |
| SCA21 | TMRM240 | (120) |
| SCA23 | PDYN | (121) |
| SCA25 | SCA25 | (122) |
| SCA26 | EEF2 | (123) |
| SCA27 | FGF14 | (27) |
| SCA28 | AFG3L2 | (124) |
| SCA29 | ITPR1 | (125) |
| SCA30 | 4q34.3-q35.1 | (126) |
| SCA31 | BEAN1 | (127) |
| SCA34 | ELOVL4 | (128) |
| SCA35 | TGM6 | (129) |
| SCA36 | NOP56 | (29) |
| SCA37 | 1p32 | (130) |
| SCA38 | ELOVL5 | (131) |
| SCA40 | CCDC88C | (36) |
| DRPLA | ATN1 | (132) |
| SCA42 | CACNA1G | (57) |
| ADCADN | DNTM1 | (133) |

*ADCADN – Autosomal Dominant Cerebellar Ataxia Deafness and Narcolepsy.*

Supplementary Data Table 1.0 All currently known ADCA disease subtypes and corresponding genes.
